# Cryopreservation of testicular tissue from Murray River Rainbowfish, *Melanotaenia fluviatilis*

**DOI:** 10.1038/s41598-020-76378-7

**Published:** 2020-11-09

**Authors:** Nicola Rivers, Jonathan Daly, Robert Jones, Peter Temple-Smith

**Affiliations:** 1 Department of Obstetrics and Gynaecology, School of Clinical Sciences, Monash University, Monash Medical Centre Level 5, Block F, Room 5.EH.30, c/o 27-31 Wright Street, Clayton, Melbourne, VIC 3168 Australia; 2 Smithsonian Conservation Biology Institute, Front Royal, VA 22360 USA; 3 Hawaii Institute of Marine Biology, 46-007 Lilipuna Rd, Kaneohe, HI 96744 USA; 4 The Aquarium Vet, Melbourne, VIC Australia; 5 Australian Frozen Zoo, Melbourne, VIC Australia

**Keywords:** Ichthyology, Conservation biology, Spermatogenesis

## Abstract

Globally, fish populations are in decline from overfishing, habitat destruction and poor water quality. Recent mass fish deaths in Australia’s Murray–Darling Basin highlight the need for improved conservation methods for endangered fish species. Cryopreservation of testicular tissue allows storage of early sperm precursor cells for use in generating new individuals via surrogacy. We describe successful isolation and cryopreservation of spermatogonia in an Australian rainbowfish. Testis histology showed rainbowfish spermatogonia are large (> 10 μm) and stain positive for Vasa, an early germ line-specific protein. Using size-based flow cytometry, testis cell suspensions were sorted through “A” (> 9 μm) and “B” gates (2–5 μm); the A gate produced significantly more Vasa-positive cells (45.0% ± 15.2%) than the “B” gate (0.0% ± 0.0%) and an unsorted control (22.9% ± 9.5%, *p* < 0.0001). The most successful cryoprotectant for “large cell” (> 9 μm) viability (72.6% ± 10.5%) comprised 1.3 M DMSO, 0.1 M trehalose and 1.5% BSA; cell viability was similar to fresh controls (78.8% ± 10.5%) and significantly better than other cryoprotectants (*p* < 0.0006). We have developed a protocol to cryopreserve rainbowfish testicular tissue and recover an enriched population of viable spermatogonia. This is the first step in developing a biobank of reproductive tissues for this family, and other Australian fish species, in the Australian Frozen Zoo.

## Introduction

Total biomass of global marine environments is reported to have declined by 49% between 1970 and 2012 mostly due to continued degradation of marine ecosystems and the impacts of overfishing^1^. For freshwater environments, however, data showing global trends are difficult to acquire, but in a study of twenty-two Australian freshwater fish species, over 90% were found to have a greater than 50% chance of extinction in the next twenty years^2^. Furthermore, in Australia’s largest freshwater system, the Murray Darling Basin, fish biomass is estimated to be only 10% of what it was prior to European settlement^3^. The Murray Darling Basin river system spans 77,000 kms and passes through four states and the Australian Capital Territory. It holds significant ecological, cultural and economic significance and is home to forty-six endemic freshwater fish, over a third of which are listed as threatened or endangered due largely to exploitation, habitat degradation and the introduction of alien species^3,4^. In addition, poor water flow and chronic drought has caused algal blooms responsible for the deaths of hundreds of thousands of fish in the Basin between 2018 and 2019^5^. This sudden loss of biodiversity will likely have implications for the genetic fitness of remaining populations that were already at risk of extinction.

Cryopreservation of cells and tissues, particularly gametes, from rare and endangered species is now used to provide long-term storage of important animal genetics in collections known as “frozen zoos”^6^. Sperm cryopreservation has been successful in many fish species including Murray cod (*Maccullochella peelii peeli*)^7^, brown trout (*Salmo trutta*)^8^, Atlantic salmon (*Salmo salar*)^9^ and the zebrafish (*Danio rerio*)^10^. While physiological differences between species has made the development of a robust method of cryopreserving fish spermatozoa challenging, particularly for endangered species where samples are often limited, sperm cryopreservation remains a common and successful method of fish biobanking. However, the seasonality of fish spawning periods means that there is sometimes limited opportunity to collect and bank mature fish gametes, and utilising cryopreserved sperm to produce offspring requires access to mature females of the same species. In this context, there is a need to expand current biobanking targets beyond sperm cryopreservation alone. Early stages of the germ line, specifically the oogonia and spermatogonia, are large, round cells found in the gonads of female and male fish, respectively. These cells have been successfully cryopreserved in various fish species including tench (*Tinca tinca*)^11^, tiger puffer (*Takifugu rubripes*)^12^, brown trout (*Salmo trutta*)^13^, rainbow trout (*Oncorhynchus mykiss*)^14^, manchurian trout (*Brachymystax lenok*)^15^, marine goby (*Asterropteryx semipuctata*)^16^, Siberian sturgeon (*Acipenser baerii*)^17^, sterlet (*Acipenser rethenus*)^18^ and Chinese rosy bitterling (*Rhodeus ocellatus ocellatus*)^19^. Spermatogonia are a flexible target for biobanking compared to spermatozoa, as these cells can be collected from a fish at any life stage, without having to target adult fish during spawning periods. In addition, the relatively simple structure of these cells has meant that similar cryopreservation methods can be applied across species. Previous studies have used broadly similar cryoprotectant formulations and cooling methods^14–16,20^ with the advantage of greatly reducing the time and resources needed to optimise an entirely new cryopreservation protocol for an endangered fish species. The application of this technique could vastly improve management strategies needed for endangered fishes in the Basin and other freshwater systems in Australia. The Australian Frozen Zoo (AFZ) is a biobanking initiative focused on the collection and storage of tissue and cells from endangered species. In its twenty-five-year history, the AFZ has accumulated over five thousand samples from Australian and exotic wildlife, however, there is little representation from fish species in the collection. In collaboration with the Australian Frozen Zoo, the aim of this study was to cryopreserve gonadal tissue from a native species of rainbowfish as a model for future biobanking initiatives in Australian fish species.

The rainbowfish family, Melanotaeniidae (order Atheriniformes), consists of ten currently recognized genera, the largest being *Melanotaenia*. Distributed across Australia, New Guinea and surrounding islands, the rainbowfish family includes several listed species including the critically endangered Running River Rainbowfish (*M. splendida. nov*) and Malanda Rainbowfish (*M. splendida. nov*) and the endangered Lake Eacham Rainbowfish (*M. eachamensis*), Slender Rainbowfish (*M. gracilis*) and Utchee Creek Rainbowfish (*M. utcheensis*)^21^. We have selected the Murray River Rainbowfish (*Melanotaenia fluviatilis*) to determine the efficacy of gonadal cryopreservation methods in an Australian fish species. The Murray River Rainbowfish is currently classified as vulnerable in the state of Victoria^22^ but is still available for research from commercial suppliers, making it an ideal model for optimization of these methods for the large Melanotaeniidae family.

Using the research of Lee et al.^14,15^ and Hagedorn et al.^16^ as a starting point for our experimental design, we have developed a cryopreservation protocol for testis tissue from *M.fluviatilis* that produces viable spermatogonial cells post-thaw. To our knowledge, this is the first time cryopreservation of gonadal tissue has been attempted in an Australian fish species as well as within the order of Atheriniformes which comprises silver-sides and rainbowfishes distributed across the globe. We have validated cell sorting methods previously described by Hagedorn et al^18^, to provide an optimised method for the cryopreservation and isolation of an enriched population of viable, target germ cells from the testis of *M.fluviatilis* for use in future applications, such as cell culture or germ cell transplantation. We see this as a vital first step towards the expansion of biobanking programs for Australian fish species.

## Methodology

### Animal husbandry and sample collection

All animal handling and experimental procedures were approved by the Animal Ethics Committee B at Monash Medical Centre (MMCB/2017/39) and conducted in accordance with the Australian Code of Practice for the Care and Use of Animals for Scientific Purposes. *Melanotaenia fluviatilis* (Aquarium Industries, Victoria, Australia) were held at 25 °C ± 1 °C on a 12:12 light–dark cycle. At the time of experimentation, fish 5.76 cm ± 1.00 cm in length and weighing 3.25 g ± 1.38 g, were humanely killed by anesthetic overdose using aquatic anaesthetic AQUI-S (Primo Aquaculture, Queensland, Australia) and death was confirmed by destruction of the brain. The gonads were removed and placed into handling medium composed of Eagles minimum essential media (EMEM, SigmaAldrich) supplemented with 5% FBS (ThermoFisher Scientific, Victoria Australia), and 25 mM HEPES (ThermoFisher Scientific; pH 7.8) and kept on ice.

### Histology and immunohistochemistry

Whole testes were fixed in 10% neutral buffered formalin (Merck, Victoria, Australia) for 48 h and processed by the Monash Histology Platform which included standard hematoxylin and eosin staining. Unstained sections were stained for Vasa using a zebrafish-specific anti-Vasa antibody (Sapphire Bioscience Pty. Ltd, New South Wales, Australia) and counter-stained with Hoechst (ThermoFisher Scientific). De-paraffinised sections were rehydrated through changes of xylene and a standard series of decreasing ethanol dilutions before antigen retrieval in 10 mM citrate buffer (pH 6), microwaved to boiling point for 10 min. Sections were rested in citrate buffer for 30 min prior to blocking with CAS Block (Invitrogen) for one hour followed by incubation with anti-Vasa antibody (1:200) in 5% BSA in PBS at 4 °C overnight. Sections were washed in PBS and incubated with secondary antibody, Alexa Fluor 488-conjugated goat anti-rabbit IgG (1:500; Invitrogen), and Hoechst nuclear counterstain (1:1000) in 5% BSA and PBS for one hour at room temperature.

Images were captured using the EVOS FL Auto 2 Imaging system (ThermoFisher Scientific) and an Olympus BX43 Upright Microscope with an X-Cite Series 120 Q laser (Lumen Dynamics). Approximate cell sizes were measured using cellSens Standard imaging software (Software version: 1.16, build 15,404, Olympus) and images were analysed in FIJI^23^ (Software version: 2.0.0-rc-69/1.52p, Image J).

### Validation of size-based cell sorting by flow cytometry

Using cell measurements taken from histological analysis as a guide, a size-based cell sorting method was developed to isolate our target spermatogonial cells. A set of five size-specific beads (16.5 μm, 10.2 μm, 7.56 μm, 5.11 μm, 3.3 μm, Spherotech, Lake Forest, IL, USA) were analysed on a FACS Aria Fusion flow cytometer (BD Biosciences, New South Wales, Australia). These sizes cover the range of cell sizes seen in the testis, with sperm heads being approximately 2–3 μm and spermatogonia being over 10 μm in *M.fluviatilis*. Due to differences in the light scattering properties of plastic beads in comparison to live cells, these bead sizes can only be interpreted as a guide of scale and not as an exact size indication for cells in suspension. Using the scatter profile produced by these beads, two gates were set: the “A” gate surrounded events in the high forward scatter region on the scatter plot, approximately 9 μm and larger to capture larger cells such as spermatogonia; the “B” gate surrounded events in a low forward scatter region, between 2—5 μm, to capture smaller germ cells such as spermatids and spermatocytes. An unstained cell suspension was then sorted through these gates and sorted cells were pelleted by centrifugation (500 g for 15mins). Images were taken of live cells in suspension using the EVOS FL Auto 2 Imaging system (ThermoFisher Scientific) and cell sizes were measured in FIJI. Samples were then fixed in 2% PFA (Thermo Fisher Scientific) for 10 min and suspended in PBS.

Aliquots of each sample (A gate, B gate and an unsorted control) were smeared onto Superfrost Plus slides (ThermoFisher Scientific), baked overnight at 37 °C and stained with anti-Vasa antibody to determine the number of Vasa-positive cells in each sample. Briefly, the slides were washed with MilliQ water to remove any salt that was present and irrigated with wash buffer (0.1% BSA in PBS) before blocking with 10% goat serum, 0.1% Triton X in PBS for 45 min. Sections were stained with anti-Vasa antibody (1:200) in PBS containing 5% BSA for 1 h at room temperature, washed with wash buffer, incubated with Alexa Fluor 488-conjugated goat anti-rabbit IgG (1:500), and counterstained with Hoechst (1:1000). Sections were imaged on the EVOS FL Auto 2 and analysed using FIJI.

### Cryopreservation protocol

This cryopreservation method was adapted from research described by Lee et al.^14,15^. Whole gonads weighing 0.0124 g ± 0.0095 g were transferred into 1.2-ml CryoTubes with 500 μl of cryomedia containing a permeating cryoprotectant, dimethyl sulfoxide (DMSO), ethylene glycol (EG), methanol or glycerol (all purchased from Merck), at concentrations ranging between 1.0 M and 2.0 M, with 0.1 M trehalose (Merck), and 1.5% BSA (Bovogen Biologicals Pty. Ltd, Victoria, Australia) in a mixed salt solution (~ 296 mOsm, pH 7.8) previously described by Lee et al.^14^. Control samples contained all components except the permeating cryoprotectant. Samples were equilibrated on ice for one hour and then cooled at a rate of -1 °C/minute in a CoolCell (Merck) in a -80 °C freezer for at least 3 h before being plunged into liquid nitrogen. Samples were held in liquid nitrogen for at least 24 h before thawing.

### Thawing and cell suspension preparation

Samples were thawed in a 30 °C water bath for 1 min. The gonad was removed and gently blotted on a Kim-wipe to remove excess cryoprotectant residue and then rehydrated in three changes of handling medium (as described under “Animal husbandry and sample collection”) for 20 min per change (60 min total). After rehydration, the testis was placed in a tissue grinder with 500 μl of PBS and crushed. The tissue grinder was washed with another 500 μl of PBS resulting in a final volume of 1 ml. The cell suspension was passed through a 40 μm nylon filter to remove any large particulates prior to flow cytometry.

### Viability assessment by flow cytometry

Cell suspensions were stained with the LIVE/DEAD Sperm Viability Kit (ThermoFisher Scientific) which included a membrane-permeating SYBR14 nucleic acid dye for detecting live cells and membrane-impermeable Propidium Iodide (PI) nucleic acid dye to detect membrane-compromised, presumably dead cells. SYBR14 was added and incubated for 5 min in the dark, followed by PI for a further 5-min incubation.

**Figure 1:**
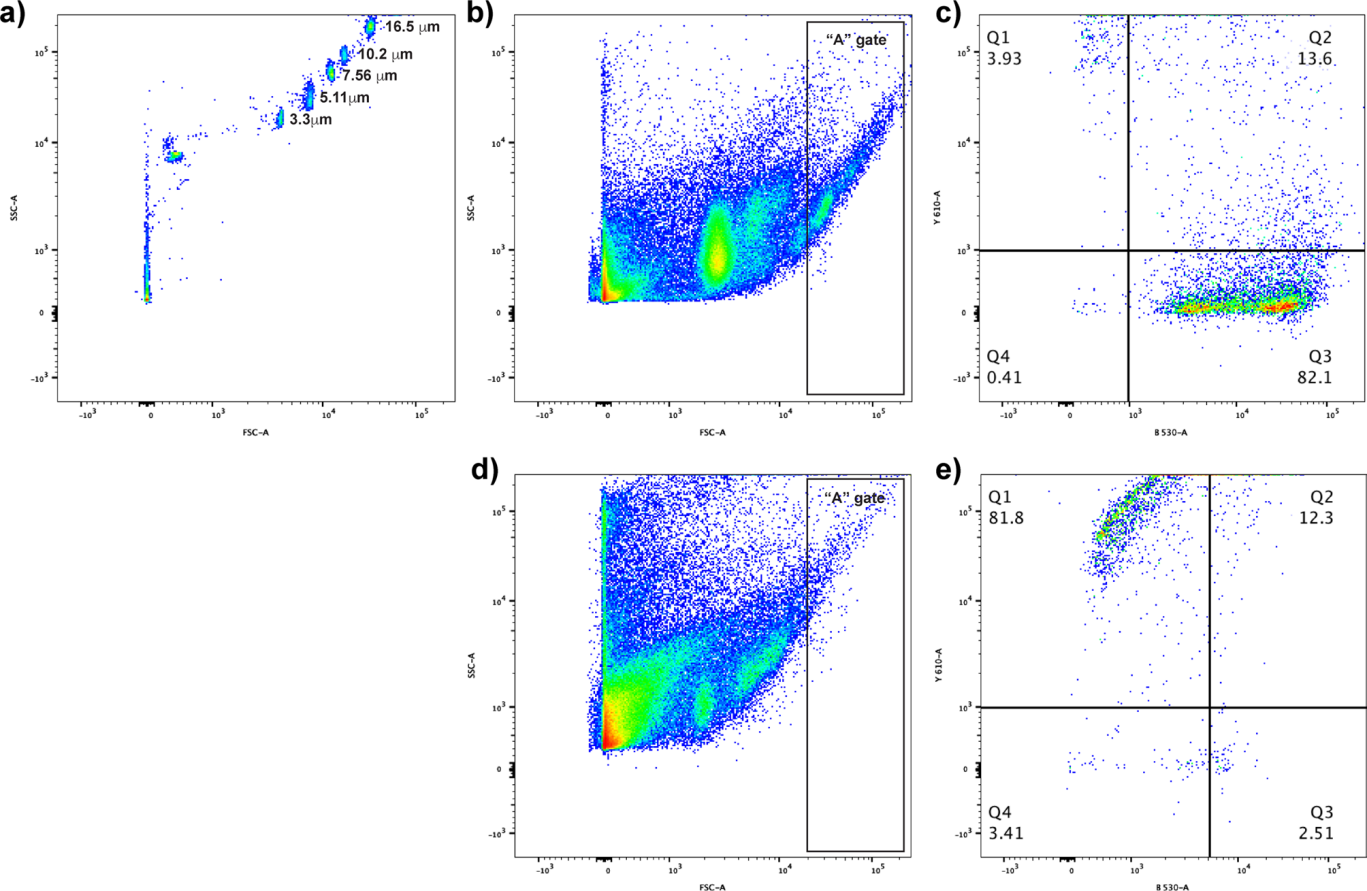
Flow cytometry scatter plots and gating method. (**a**) Analysis of size-specific beads shows five distinct clusters. (**b**) A gate is set to capture events from the 9 μm measurement and above. (**c**) Events detected in this region are replotted to determine SYB14 and PI absorbance. Events in the Q3 region are SYB14 positive and PI negative and therefore viable. In samples treated with a negative control (**d**), the majority of events falls in the Q1 region, with only propidium iodide detected (**e**).

Prior to the assessment of experimental samples, the sized beads (Spherotech) were analysed on the FACS Aria Fusion flow cytometer. Using these beads as a guide, a gate was set for the approximate size of the spermatogonial cells based on our own histological analysis of this species and previous publications on fish in general^24^. An unstained control and two single stain controls (PI only or SYBR14 only) were included with the experimental samples in the analysis. The sample used for the PI-only control was flash frozen in liquid nitrogen three times to ensure a high percentage of dead cell to provide an adequate count for PI staining. Flow cytometry output was analysed in FlowJo^TM^^25^. Events captured by the gate were analysed for SYBR14 and PI spectra and divided into quartiles based on the absorbance of single stain controls (Fig. 1).

### Statistical analysis

Statistical analysis was performed using GraphPad Prism version 8.1.2 for MacOS, GraphPad Software, La Jolla California USA, www.graphpad.com. Data is presented as mean ± standard deviation, with a p-value less than 0.05 considered statistically significant.

For cell gating data, the proportion of cell sizes in live cell suspensions in each treatment group was analysed using a chi-square. The percentage of Vasa-positive cells in the unsorted sample and the “A” gate was analysed using an un-paired t-test; data for the “B” gate was excluded as no Vasa-positive cells were detected.

For percentage viability data assumptions for normality and variance were met using the Shapiro–Wilk test and the Brown-Forsythe test, respectively. Following this, treatment groups were compared by one-way ANOVA and Tukey’s post hoc test.

## Results

### Histological examination of the testes

**Figure 2:**
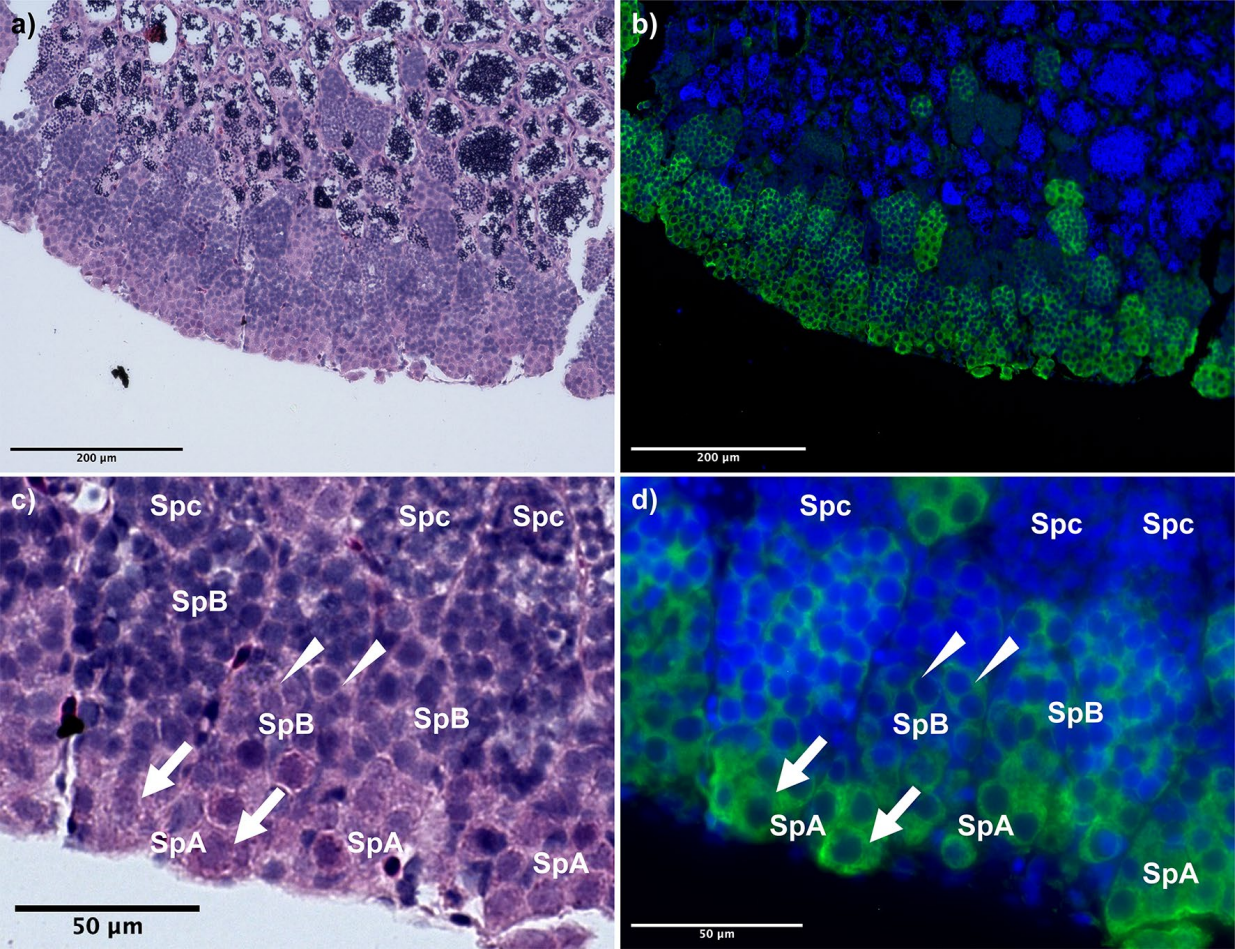
Histology of the testis of *M. fluviatilis.* Overview of outer region of the testis stained with hematoxylin and eosin (**a**) and germ line marker Vasa and Hoechst nuclear counter-stain (**b**). (**c**) Large, hypo-chromatic type A spermatogonial cells line the boundary of the testis (SpA, arrows). Type B spermatogonia are smaller in size and have a darker staining nucleus (SpB) and divide incompletely sometimes appearing to have two nuclei (arrow heads). Spermatocytes (Spc) are smaller still in comparison to type B spermatogonia. (**d**) Type A spermatogonia are strongly Vasa-positive with faint staining nuclei, whereas type B spermatogonia stain strongly for both Vasa and Hoechst. Spermatocytes have little to no Vasa staining. Bar: A-B = 200 μm, C-D = 50 μm.

*Melanotaenia fluviatilis* has paired, partially-fused testes attached to the dorsal body wall that converge to form a single opening at the urogenital pore. Testes are highly organized, with large early-stage germ cells predominantly located around the periphery and mature spermatozoa deposited in a central lumen running through the length of each testis. Spermatogenesis occurs within spermatocysts as a series of synchronized divisions and differentiation steps. Based on histological assessment in other fish species, spermatogonia typically appear as large, lightly-stained cells, while the later spermatogenic stages appear as smaller cells with more tightly packed chromatin and hence darker nuclear staining^24^. This pattern was confirmed in *M.fluviatilis*, which showed light nuclear staining and strong anti-Vasa staining in the large spermatogonial cells located at the periphery of the testis (Fig. 2). In contrast, early stage spermatocytes had weak to no Vasa staining, and no Vasa staining was observed by the spermatid stage (Fig. 2). Approximate dimensions of strongly Vasa-stained cells ranged between 10 and 18 μm. This confirmed that spermatogonial cells in *M.fluviatilis* are comparatively large cells that stain strongly for Vasa. These two characteristics were then used to develop and validate an isolation method for spermatogonia, our target cells, using flow cytometry.

### Flow cytometric analysis of testis cell suspensions

**Figure 3:**
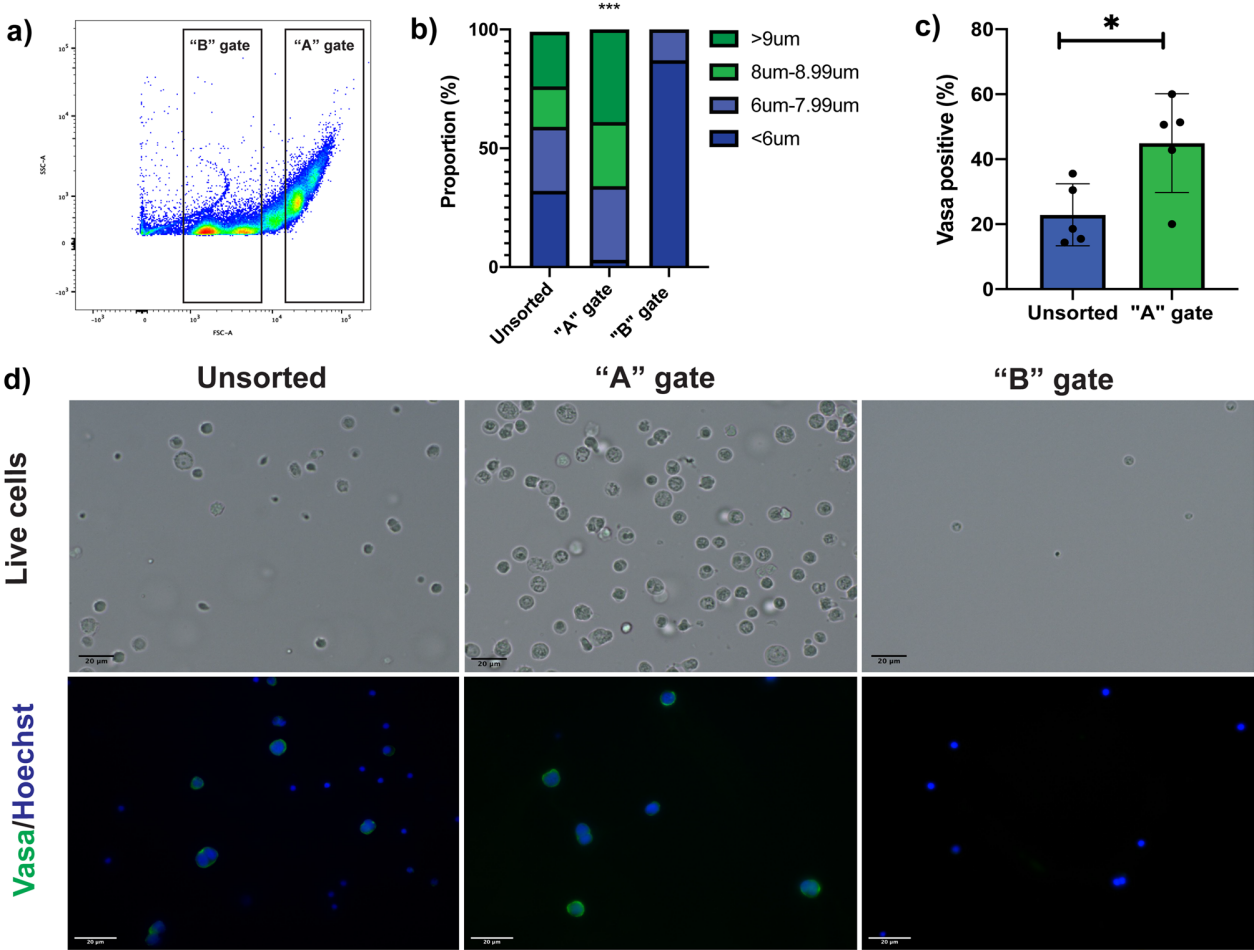
Enrichment of Vasa-positive spermatogonial cells after sorting a cryopreserved testis cell sample. (**a**) A cell suspension is analysed by the flow cytometer and two gates were set, the “A” gate and the “B” gate. (**b**) Measurements of cell sizes in the unsorted sample, “A” gate and “B” gate showed the “A” gate captures a significantly higher proportion of large testis cells > 9 μm (Chi-square, *p* < 0.001). (**c**) Staining cell samples from each group showed the “A” gate had a higher percentage of Vasa-positive cells compared to an unsorted sample (un-paired T-test, *p* = 0.0251), no Vasa-positive cells were detected through the “B” gate thus this data was not included in analysis. (**d**) Images of cells from all three treatment groups, including live cells in suspension and fixed cells stained with Vasa and Hoechst. Cell sizes in stained images differ in comparison to live images due to the dessication of cells during fixation to slides, a figure to support this is inlcuded in the supplementary material.

Side scatter (SSC) and forwards scatter (FSC) output for the size-specific beads show five clear bead populations as shown in Fig. 1. When a testis homogenate was analysed (Fig. 3a), the A gate, which captured events approximately 9 μm and above, produced a cell sample containing a larger proportion of large germ cells (> 9 μm) compared to an unsorted sample and the “B” gate (*p* < 0.001; Fig. 3b). Cells decreased in size during desiccation (supplementary Fig. 1) but subsequent staining with Vasa showed the “A” gate captured 45.0% ± 15.2% Vasa-positive cells which was significantly higher compared to an unsorted sample which contained 22.9% ± 9.5% Vasa-positive cells (*p* = 0.0251; Fig. 3c,d). This demonstrated that the flow cytometry gates set using the scatter plots of the sized beads captured a higher proportion of spermatogonial cells and provided an effective means of isolating an enriched population of spermatogonial cells from cryopreserved *M. fluviatilis* testis cell homogenates.

### Cryoprotectant optimization

**Figure 4:**
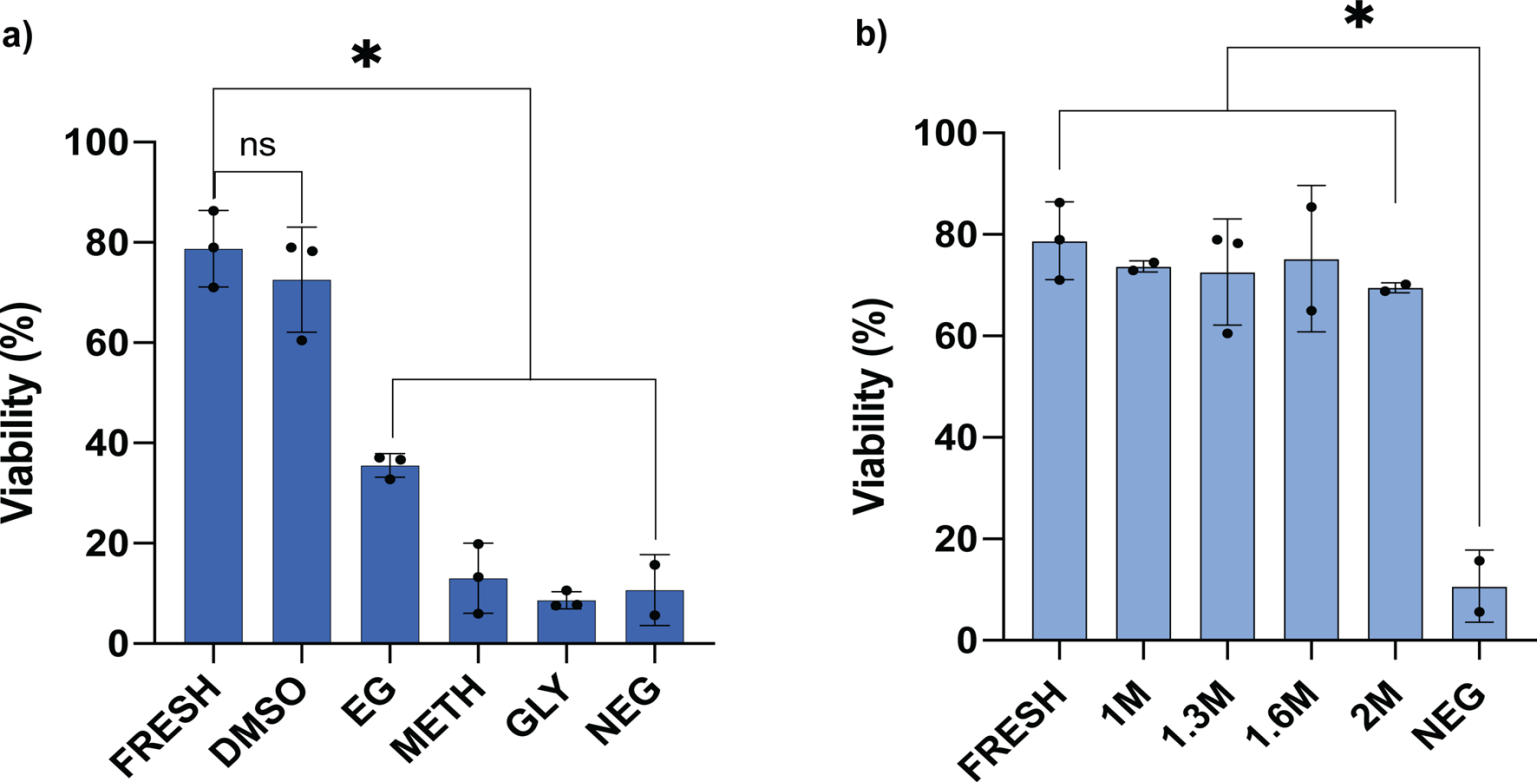
Optimization of testis tissue cryomedia in *M.fluviatilis*. (**a**) Viability of large testis cells detected in the gonad cell suspension after cryopreservation in four different permeating cryoprotectants-dimethyl sulfoxide (DMSO), ethylene glycol (EG), methanol (METH) and glycerol (GLY) (*p < 0.0001, n = 17). (**b**) Comparison of large testis cell viability after cryopreservation with DMSO at different concentrations (*p < 0.0008, n = 15).

Comparison of all four permeating cryoprotectants at 1.3 M showed that DMSO was the least toxic to the spermatogonia with a post-thaw viability of 72.6% ± 10.5%, which was significantly higher than EG (35.5% ± 2.4%, *p* = 0.0004), methanol (13.1% ± 7.0%, *p* < 0.0001), glycerol (8.6% ± 1.7%, *p* < 0.0001) and the cryoprotectant-free negative control (10.7% ± 7.1%, *p* < 0.0001; n = 17, Fig. 4a). Subsequent assessment of samples cryopreserved in DMSO at varying concentrations (1.0 M, 1.6 M and 2.0 M), showed no significant difference in viability in relation to these concentrations compared to fresh controls (78.8% ± 7.6%, *p* > 0.8373; n = 14, Fig. 4b). This suggested that DMSO concentration within the range of 1.0–2.0 M has a relatively low level of toxicity to the larger testis cells such as spermatogonia and provided a high degree of protection during cryopreservation. A comparison of cells per gram of tissue was not significantly different between fresh and cryopreserved tissue (*p* = 0.5274, Kruskal–Wallis test; Supplementary Fig. 2) indicating that cryoprotectants did not cause significant cell lysis that could have been excluded from subsequent analysis.

## Discussion

Here we report successful cryopreservation of testis tissue in the species *M. fluviatilis* and have developed and validated a protocol to isolate spermatogonia for future downstream applications such as germ cell transplantation in rainbowfish and other fish species. The cryopreservation of less differentiated cells such as spermatogonia, oogonia and primordial germ cells presents an alternative biobanking method for species in which it may be difficult to acquire mature gametes such as spermatozoa. Successful cryopreservation of rainbowfish gonadal tissue provides a readily available method for the preservation of a reproductive and genetic resource for rainbowfish and other Australian fish species^26^.

The viability of spermatogonia was highest following cryopreservation with 1.3 M DMSO and 0.1 M trehalose in 1.5% BSA and a mixed salt solution. This was consistent with previous studies in rainbow trout^14^ and marine goby^16^ in which DMSO also resulted in the highest large cell viability. This confirms that DMSO is broadly successful as a cryoprotectant and should be considered for future conservation use in other rainbowfish species such as the related, endangered, Lake Eacham Rainbowfish. It needs also to be tested using more distantly related fish species in the Murray–Darling Basin such as the iconic Murray cod. The ability to cryopreserve gonadal tissues also eliminates the possible challenges of obtaining gametes or embryos, imposed by specific breeding conditions and seasonal breeding activity which are often difficult to predict due to decreases in fish populations and changing climatic conditions. The ability to secure the reproductive potential and genetics of fish during any life stage is of great benefit to future fish conservation strategies.

The use of biobanking and the establishment of “Frozen Zoos” is not an alternative to in situ conservation strategies but rather a supplemental strategy to minimize the risk of losing genetic diversity. Further techniques such as in vitro differentiation^27^ or germ cell surrogacy^28^ are required to generate new animals from frozen tissue, particularly when the cells frozen are undifferentiated and not able to immediately produce offspring. In this study, we have validated a cell isolation method in our target species using flow cytometry. While this method was used specifically to analyze the viability of large testis cells in a testis cell suspension, it has a dual purpose in that we can now produce a sample of concentrated spermatogonial cells for future downstream applications. Here, we have included both type A and type B spermatogonia in our analysis as under the right circumstances both cell types could produce viable spermatozoa in vitro. Whole testis cell suspensions have been successfully transplanted into surrogates via germ cell surrogacy^17,19,29,30^, but an enriched sample of spermatogonial cells, particularly type A spermatogonia, is preferred as this can improve the success rate of surrogacy experiments^31^. There are several methods for isolating spermatogonial cells from a gonadal cell suspension including gradient centrifugation^32,33^ and elutriation^34^, fluorescent activated sorting (FACS)^14,35,36^ and magnetic activated sorting (MACS)^37–39^. Gradient based sorting, while the most cost effective, may be difficult to use in species with smaller gonads and a low spermatogonia cell number if bands are not easily identified in the centrifuged column. Tissue from endangered species is often limited and may therefore benefit from more specific methods such as FACS, but this method usually requires a large number of cells which may not be possible in some endangered species. In addition, in the absence of a broadly applicable cell surface marker to detect live spermatogonia, this is often restricted to transgenic species^14,36^. Similarly, MACS faces the same issues regarding antibody development to detect spermatogonia, although success has been reported in some species^37–39^. Ultimately, an isolation method that is specific but does not require the use of transgenic fish or antibodies is what will be most applicable in endangered species.

Using flow cytometry, cell specific light-scattering profiles can be detected allowing for the identification of unique populations. Using these profiles, spermatogonia have been successfully isolated in rainbow trout^31,36,40^, Japanese char^31^, masu salmon^31^ and Pacific blue fin tuna^35^. Across multiple species, Vasa-positive spermatogonia have been detected in the high forward scatter region which correlated with their large size^31,35^. We adapted the size-based flow cytometry method previously reported by Hagedorn et al^16^ in a marine goby, as a means of assessing large cell viability in *M.fluviatilis*. In addition, we have developed an immunocytostaining protocol to detect anti-Vasa antibody in cells sorted through the gate to determine the efficiency of the cell size gating method for spermatogonia specifically. We found that gating testicular cell suspensions in the high forward scatter region produced an enriched sample of Vasa-positive, spermatogonial cells. This was consistent with previous studies thereby providing further evidence that size-based methods of gating spermatogonial cells could be a broadly applicable, non-transgenic means of isolating these cells across many species with relatively little need for optimization.

At present, the greatest challenge in applying gonad cryopreservation to produce live offspring is the development of suitable surrogates in which to perform germ cell transplantation for the production of gametes. Continued investigation by specialist groups has enabled the development of these procedures in some species (e.g.^27,30,41,42^), but a great deal more research is now required to realise the full potential of gonad cryopreservation and germ cell surrogacy in the majority of threatened fish species. However, gonad cryopreservation remains an extremely powerful conservation tool, given that it can be used to secure useable genetic material from almost any life stage regardless of reproductive seasonality. Moreover, the relatively simple cryopreservation procedure used to cryopreserve fish gonads requires minimal equipment and training, making them suitable for field application^16^, particularly in situations where liquid nitrogen use would be difficult. While the development and refinement of transplantation and surrogacy methods for fish will be crucial to the future utility of cryopreserved spermatogonial cells, the cryopreservation of gonadal tissues as outlined in the present study can still be undertaken in the present so that broad genetic diversity is available for future recovery efforts. With the challenges confronting fish species globally, cryopreservation will be an important strategy to preserve genetic material, and gonad cryopreservation provides an important new tool to complement sperm cryopreservation for securing the biodiversity of threatened fish species worldwide.

Our findings demonstrate that cryopreservation of gonadal tissue is a viable option for the preservation of rainbowfish genetic resources for current and future conservation programs. The application of this simple, effective method for the biobanking of fish reproductive tissue will be vital to the management and protection of vulnerable and endangered fish species in Australia and globally.

## Supplementary information

Supplementary Figures.

## Data Availability

The datasets generated during and/or analysed during the current study are available from the corresponding author on reasonable request.
