# Supplementary material for: Cryopreservation of testicular tissue from Murray River Rainbowfish, Melanotaenia fluviatilis
Source: Sci Rep. 2020 Nov 9;10:19355. doi: 10.1038/s41598-020-76378-7 (PMC7653925; doi:10.1038/s41598-020-76378-7)
Supplement: Supplementary file 1 — Supplementary Figures. [file 41598_2020_76378_MOESM1_ESM.docx]

**Title-** Cryopreservation of testicular tissue from Murray River Rainbowfish, *Melanotaenia fluviatilis*

**Running title:** Cryopreservation of testicular tissue from Murray River Rainbowfish*.*

**Authors**

***Nicola Rivers^1,5^, Jonathan Daly^2,3,5^, Robert Jones^4^ & Peter Temple-Smith^1,5^**

**Affiliations**

^1^Department of Obstetrics and Gynaecology, School of Clinical Sciences, Monash University, Melbourne, VIC, Australia

^2^Smithsonian Conservation Biology Institute, Front Royal, VA, 22360, United States of America

^3^Hawaii Institute of Marine Biology, 46-007 Lilipuna Rd, Kaneohe, HI, 96744, United States of America

^4^The Aquarium Vet, Melbourne, Victoria, Australia

^5^Australian Frozen Zoo, Melbourne, Victoria, Australia

**Corresponding Author**

Nicola Rivers

Mailing address:
Monash Medical Centre Level 5, Block F, Room 5.EH.30

c/o 27-31 Wright Street

Clayton VIC 3168

Australia

E: [Nicola.rivers@monash.edu](mailto:Nicola.rivers@monash.edu)


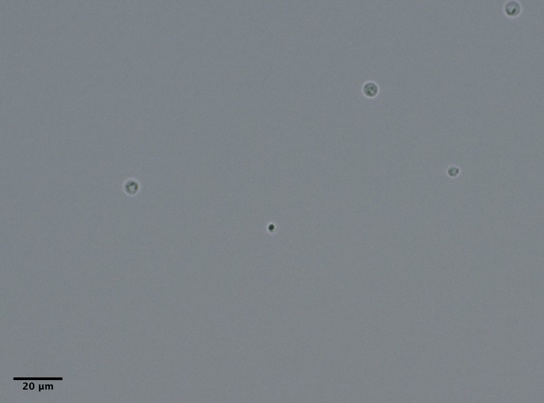

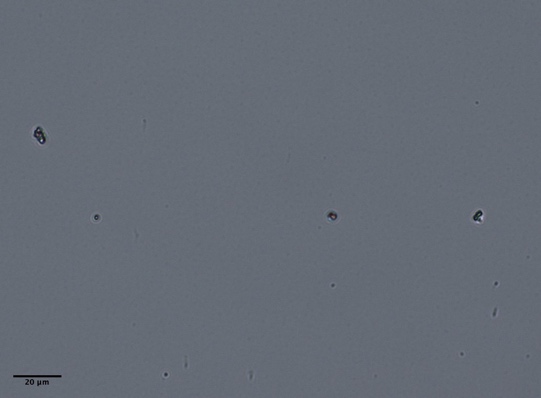

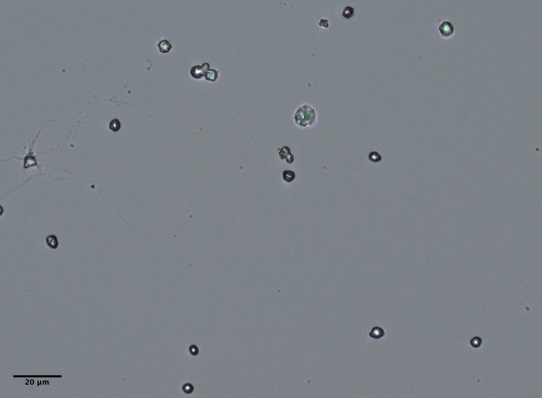

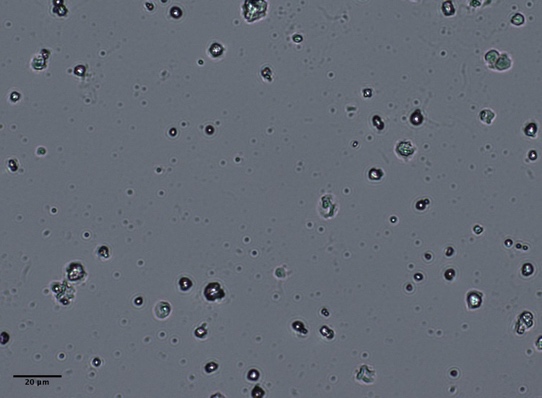

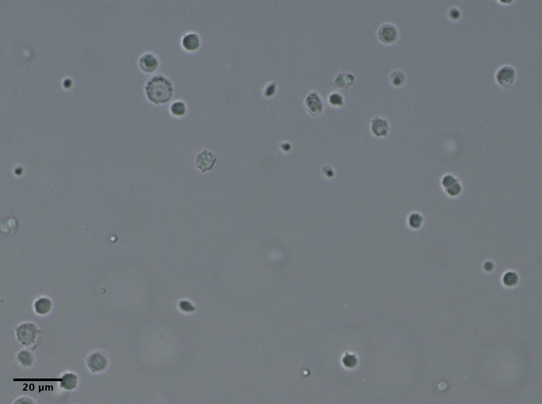

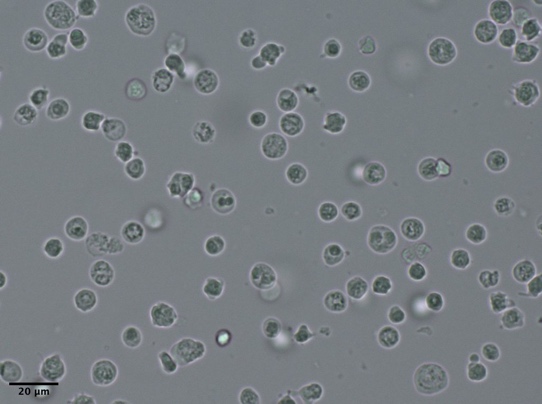


A

B

C

D

E

F

**Supplementary figure 1**. Visual comparison of cell sizes in live (A-C) compared to dried (D-F) cell samples in an unsorted sample (A and D), the “A” gate (B and E) and the “B” gate (C and D).

**Supplementary figure 2.** Number of cells per gram in fresh and cryopreserved tissue (p=0.5274, Kruskal-Wallis test).
